# Lower extremity MRI: are their requests always appropriate in France?

**DOI:** 10.1007/s00330-025-11402-w

**Published:** 2025-02-04

**Authors:** Adeline Degremont, Valérie Lindecker-Cournil, Catherine Bisquay, Valérie Ertel-Pau, Pierre Gabach, Sophie Lecocq Teixeira, Jean-Baptiste Pialat, Pierre-Alain Jachiet, Louis Boyer, Marie Faruch-Bilfeld

**Affiliations:** 1 Haute Autorité de Santé, Data Mission, La Plaine Saint-Denis, France; 2 Haute Autorité de Santé, Guidelines Department, La Plaine Saint-Denis, France; 3 IMALO, Medical Imaging Center, Maxeville, France; 4 Université Claude Bernard Lyon 1 and Hospices Civils de Lyon, Department of Radiology, Lyon, France; 5 University Hospital Centre Clermont-Ferrand, Radiology Unit, Clermont-Ferrand, France; 6 University Hospital Centre Toulouse, Radiology Unit, Toulouse, France

**Keywords:** Magnetic resonance imaging, Lower extremity, Knee, Practice patterns, physicians’, Chronic pain

## Abstract

**Objectives:**

The primary aim is to assess current lower extremity MRI requests’ relevance with a secondary focus on the knee.

**Materials and methods:**

Using data from the National Health Data System (SNDS), we conducted an observational study of adults (18+) who underwent lower extremity MRI between July 1 and December 31, 2021. This study included analyzing medical consultations and imaging procedures (particularly X-rays) in the 6 months before and after the index MRI, as well as medical procedures and hospitalizations related to knee procedures within 6 months post-MRI.

**Results:**

During the study period, 779,721 adults underwent lower extremity MRI, marking a 76% increase compared to a previous study conducted in 2012. General practitioners requested MRI in 70.5% of cases, often as the primary imaging modality. Notably, 52.1% of patients had not undergone lower extremity X-rays in the 6 months preceding MRI, and 13% underwent at least two MRI examinations within a year. Focusing on the knee, most patients (80%) did not undergo any outpatient medical procedure or hospitalization involving the knee within the 6 months post MRI.

**Conclusion:**

In France, lower extremity MRI, particularly knee MRI, is frequently used as a first-line imaging procedure, unlike what is recommended.

**Key Points:**

***Question*** *How often are requests for lower extremity MRI examinations appropriate?*

***Findings*** *Lower extremity MRI is often performed as a first-line imaging procedure, even though it is not recommended.*

***Clinical relevance*** *The study findings underscore the importance of disseminating guidelines regarding lower extremity MRI appropriateness to increase its availability for appropriate purposes, thereby improving patient care.*

## Introduction

Magnetic resonance imaging (MRI) today enables diagnostic evaluation for many clinical indications, including lesion detection, characterization, functional assessment, and response to treatment. The main advantages of MRI are that it is non-irradiating and non-invasive. However access to MRI can take a long time in France: a study commissioned by the National Union of the Medical Technology Industry (Syndicat national de l’industrie des technologies médicales) showed that in 2018, the estimated waiting time for an MRI scan in oncology was 32.3 days [1].

According to international guidelines [2–7], lower extremity MRI is not a first-line imaging: its request should be discussed after the X-rays have been performed. As lower extremity MRI mainly concerns the knee, according to clinicians’ feedback in France, the National Authority for Health (Haute Autorité de Santé [HAS]) and the National Professional Council for Radiology and Medical Imaging (G4) published two guidelines in 2022 on the relevant imaging in cases of non-traumatic knee pain or after knee trauma in adults [8]. In the case of non-traumatic knee pain, the main recommendations are:

- carry out a thorough history and clinical examination before any knee imaging;
- knee X-rays are the first-line imaging procedure;
- MRI should not be performed as a first-line imaging procedure;
- imaging should not be repeated in the event of a new episode of knee pain in a patient with a known pathology (e.g., osteoarthritis) and usual symptomatology.

A review of the practice showed a steady increase in the use of lower extremity MRI in France [9]. According to the analysis of patient care 3 months before and after the request for a lower extremity MRI by a general practitioner (GP) in the first half of 2012, MRI was not always relevant. In fact, this study showed that 61% of patients did not have a prior X-ray (recommended before MRI), and 6% underwent a repeat MRI examination.

The main objective of our observational study is to establish an up-to-date overview of lower extremity MRI practices almost ten years after this study, with a secondary focus on the knee.

## Materials and methods

This study was authorized by Decree 2016-1871 of December 26, 2016, on the processing of personal data in the National Health Data System (Système National des Données de Santé [SNDS]) and Articles 1461-13 and 14 of French law. The HAS, which has permanent regulatory access to SNDS data, did not require the consent of the participants or the approval of the institutional review board.

The study was based on the SNDS, which is the largest database of reimbursement claims in France [10, 11]. It contains individual data linked anonymously to an individual’s national insurance number for all healthcare expenditures reimbursed by the National Health Insurance. It includes reimbursements of outpatient healthcare consumption and data from the national hospital discharge database, i.e., data on over 99% of the French population. More specifically, the SNDS contains socio-demographic data (age, sex, and region), information on outpatient consultations (e.g., GP consultations), medications (e.g., drug dispensing), medical procedures (e.g., imaging), hospital admissions and inpatient hospital procedures with corresponding dates of care. All this data could be linked to create a longitudinal record of the patient’s healthcare pathway. However, clinical information such as reasons for consultation, therapeutic indications, and imaging reports are not recorded and therefore could not be used in this study. Nevertheless, the comprehensive nature and the near completeness of the database make it a valuable resource for studying the care pathways of patients who have undergone a lower extremity MRI.

The study population included all adults (aged 18 and over) who underwent at least one lower extremity (pelvis, hip joint, leg, knee, ankle, or foot) MRI scan (with or without injection of contrast agents) between July 1 and December 31, 2021. We excluded MRIs performed in emergency units. We evaluated the distribution of medical specialties of the physicians requesting the MRI. As there is no encoding variable in the SNDS to identify these specialist physicians, they were approximated with the physicians consulted in the 3 months preceding the index MRI (the first MRI during the study period). Their medical specialties were classified into five categories: GP, orthopedic surgeon, rheumatologist, physical medicine and rehabilitation (PMR) physician, or other medical specialty. Each patient was followed from 6 months before the date of the index MRI until 6 months after. During the follow-up period, several variables were analyzed: consultations/teleconsultations with a GP, a rheumatologist, an orthopedic surgeon, or a PMR physician, as well as imaging procedures (X-ray, joint ultrasound, lower extremity MRI, CT scan) performed on an outpatient basis or in hospital (private or public). As we wanted to focus on the knee, we also analyzed medical procedures on the knee (joint puncture, immobilization, drainage, infiltration) and hospitalization for knee surgery (meniscectomy, cruciate ligament repairment, knee replacement, other surgery by arthroscopy) in the 6 months after index MRI. It should be noted that it is not possible to know the precise location of the lower extremity MRI procedure.

The list of procedures is available in the supplementary materials. For these variables, we calculated the proportion of patients who received care in the 6 months before and after the index MRI and the distribution of time (in days) between care and the index MRI (before and after). These statistical analyses were performed using SAS Enterprise Guide Version 8.3. In accordance with data protection and French regulations, the authors cannot make public data from the National Health Data System (SNDS). However, all the programs carried out as part of this study are documented here: https://has-sante.pages.has-sante.fr/public/programmes-snds/index.html.

## Results

In the second half of 2021, in France, a total of 779,721 adults (aged 18 or older) underwent (at least) one lower extremity MRI. The median age of patients (at index date) was 50 years (IQR: [36–61]). The male/female distribution was balanced (49.2% men).

**Fig. 1:**
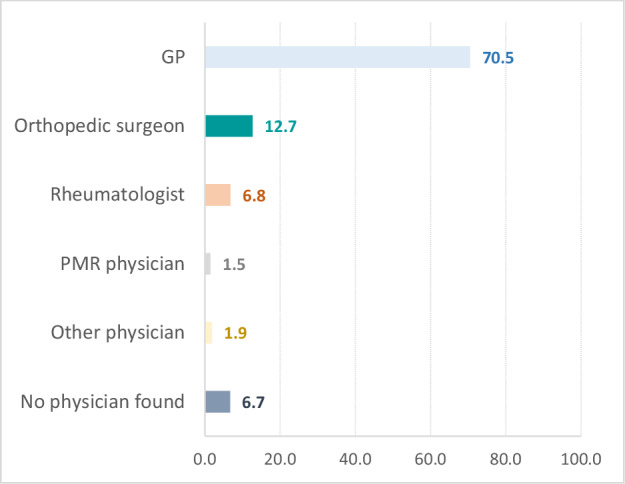
Distribution of physicians requesting lower extremity MRI (approximated by visits in the 3 months prior to MRI). PMR, physical medicine and rehabilitation; No physician found, no physician consulted in the 3 months preceding the index MRI

According to our definition (see “Materials and Methods”), the GP was the physician requesting the MRI in 70.5% (549,076/779,721) of cases (see Fig. 1).

Among the study population (*N* = 779,271), in the 6 months prior to the index MRI, almost all patients (93%, *N* = 727,701) had at least one consultation with a GP. Most patients (74%, *N* = 575,894) did not have a specialist consultation (orthopedics, rheumatology, PMR); 17% (*N* = 129,596) of patients had an orthopedic consultation, 10% (*N* = 75,184) a rheumatology consultation, and 2% (*N* = 16,873) had a PMR consultation. These consultations took place within a median period of 35 days (≈ 1 month) before the index MRI.

Within 6 months after the index MRI, 87% (*N* = 681,118) of patients had at least one consultation with a GP, within a median period of 18 days (≈ 3 weeks) after the index MRI. Half of the patients (*N* = 403,749) consulted a specialist, within a median period of 28 days (≈ 1 month) after the index MRI; 40% (*N* = 311,522) had an orthopedic consultation, 14% (*N* = 112,397) a rheumatology consultation, and 3% (*N* = 25,112) a PMR consultation.

In the 6 months prior to the index MRI, half of the patients (52%, *N* = 406,022) had not had a lower extremity X-ray (pelvis, hip joint, thigh, leg, knee, ankle, or foot).

In the 6 months prior to the index MRI, 3% (*N* = 26,432) of patients had undergone at least one previous lower extremity MRI, within a median period of 121 days (≈ 4 months,) and in the following 6 months, 11% (*N* = 82,226) had undergone another lower extremity MRI, within a median period of 42 days (≈ 6 weeks). A total of 13% (*N* = 103,950) of patients had undergone at least one MRI in addition to the index MRI in the 6 months either before or after the procedure.

Focusing on the knee, most patients (80%, *N* = 623,113) did not undergo any outpatient medical procedure or hospitalization involving the knee in the 6 months following the index MRI.

**Fig. 2:**
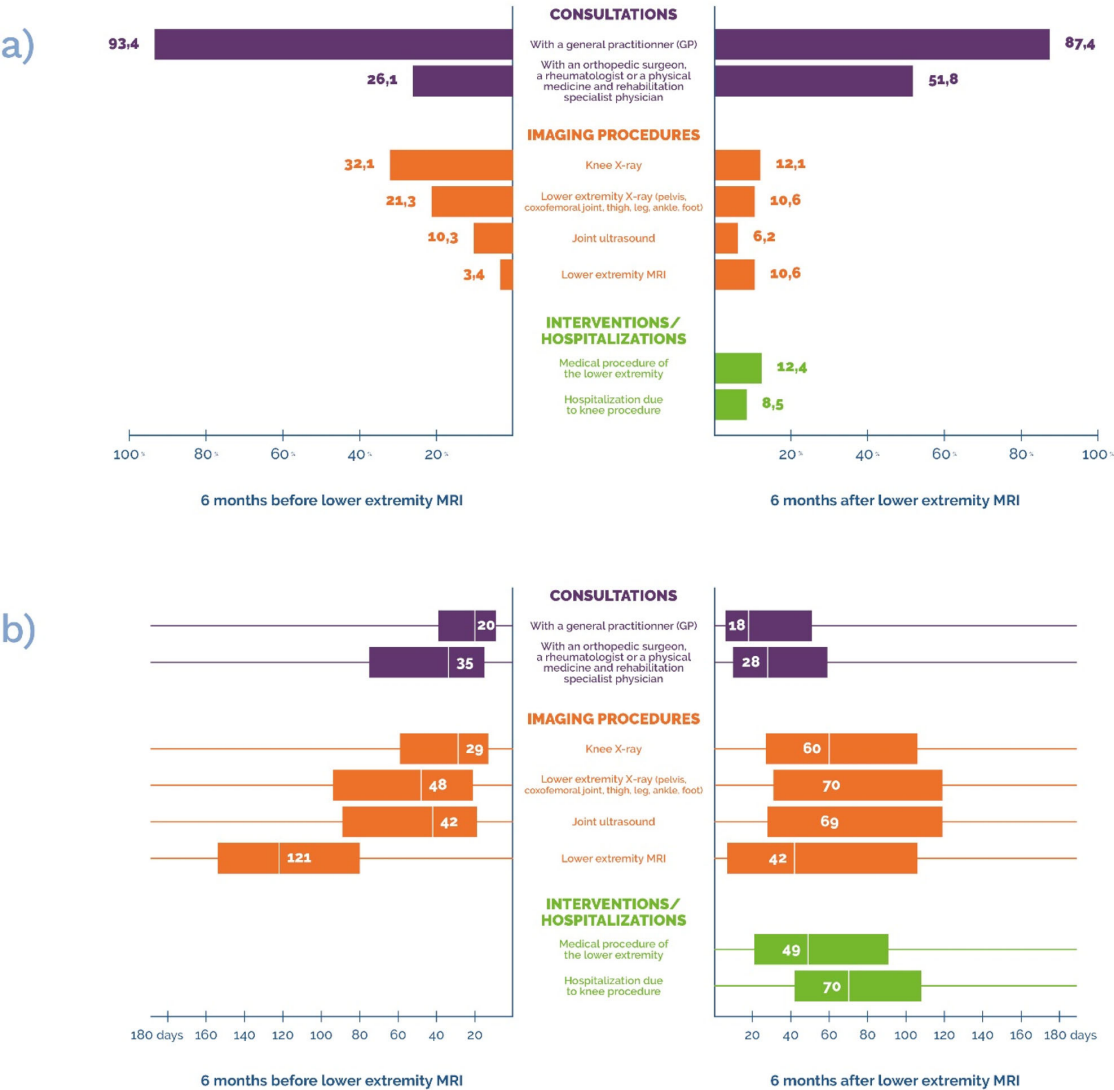
Description of healthcare received by patients in the six months prior to lower extremity MRI (left) and after lower extremity MRI (right): **a** Proportion of patients that received healthcare; **b** boxplot of the period (in days) between healthcare and index lower extremity MRI. For example: 32.1% of patients had a knee X-ray in the 6 months prior to lower extremity MRI, within a median period of 29 days before MRI

The main results regarding patients’ outcomes are summarized in Fig. 2.

## Discussion

We conducted a study using SNDS data to analyze the before-and-after (6-month) healthcare pathway of adults who had undergone a lower extremity MRI in the second half of 2021 in France. This study updates the National Health Insurance Fund (CNAM) data from 2012 on the use of healthcare before and after lower extremity MRI. It shows that the number of MRIs has risen sharply in almost 10 years (443,242 in the first half of 2012, to 779,721 in the second half of 2021, an increase of 76%). MRI was the first-line examination for at least half of the patients (52%), even though it is not recommended.

These results raise the question of the appropriateness of lower extremity MRI indications. Compared with the 2012 results observed by the CNAM, practices have not really changed. Firstly, they found that 61% of patients had not had lower extremity X-ray in the 3 months preceding the MRI. The follow-up in their study (3 months before) is shorter than ours (6 months before) and could explain why the proportion of patients without a prior X-ray observed in their study is higher than in our study. This may also be explained by the fact that they only focused on patients whose GP was the presumed requesting physician (1). However, the physician requesting the MRI is the GP for 70.5% of patients in our study, which means that the results are easily comparable. Furthermore, the CNAM observed that, among patients whose GP was the presumed requesting physician, repeated lower extremity MRI examinations were performed for 6% of patients in the 3 months preceding or following the index examination. In our study, we may also wonder whether MRI is not too frequently repeated. In fact, in the 6 months preceding or following the index MRI, 13.3% of patients had at least one other lower extremity MRI: 3.4% of patients before the index MRI and 10.6% after. Regarding the knee, the CNAM observed that less than 10% of patients underwent knee-related medical procedures within 3 months of lower extremity MRI. In our study, 20% of patients had medical procedures within 6 months of MRI. The lack of clinical data makes it difficult to evaluate the appropriateness of MRI in these patients.

Apart from the 2012 CNAM study, we have not found any other study about the relevance of lower extremity MRI or knee MRI in France. A survey carried out in 2020 by the Académie Nationale de Médecine among 260 patients seen consecutively on the same day at an osteoarticular imaging center showed that 70% of MRI requests were incomplete, with the reason for the request specified for only 14% of patients [12]. The authors underline the importance of integrating the obligation to provide sufficient clinical information into e-prescription software. Internationally, a recent systematic review of the literature concerning the appropriateness of diagnostic imaging found that for MRI of extremities, the overall appropriateness rate was 66% (*n* = 352/535) [13]. It is worth noting that the four underlying studies varied in sample size (55–300) and results (55–83%). One of the studies is focused on the knee; based on the availability of an electronic medical record (EMR) for every MRI performed in the Elche Health Department in Spain, the authors found that in 45% of cases (*N* = 135/300) the MRI prescription was inappropriate [14]. The main situations of inappropriateness included the use of MRI as the initial imaging technique. The inappropriateness of knee MRI has also been studied in other countries and has shown that practices could be improved [15–17]. In the USA, George et al, found that 22.8% of MRI examinations for non-traumatic knee pain realized in 2010 among Medicare and private insurance patients had been performed without prior radiography in the same calendar year [15]. In a study conducted in Atlanta (Georgia), in 2019, Gonzalez et al observed that of 196 MRIs (over an 18-month period), 57% (108 knees) had “usually appropriate” (i.e., 7-9) and 43% (8 knees) had “usually not appropriate” (i.e., 1-3) appropriateness criteria scores (*p* > 0.1) [16]. In Italy, in 2016, a panel of experts compared a set of guidelines with data from 400 patients who underwent previous knee MRI and found that almost 21% of prescriptions were totally inappropriate and 18.8% were uncertain [17]. The diffusion of guidelines could improve these practices. For example, a recent study by the North Bristol NHS Trust in the United Kingdom showed that the number of patients who underwent an MRI of the knee without a prior plain radiograph was 55/118 (47%) compared with 14/69 (20%) after the definition and dissemination of a referral pathway for a knee MRI [18]. In Canada, the eHealth Center of Excellence in collaboration with clinicians showed that the rate of ordering X-rays as the proper initial imaging scan for patients presenting with knee pain has steadily increased by 10% over the year for users of the eReferral platform (an integrated clinical decision support tool for diagnostic imaging requests) compared to a decrease of 7% for those using fax [19].

The main strength of our study is the use of a database covering more than 99% of the French population, which enabled us to analyze all lower extremity MRI examinations reimbursed in France. The results call into question the appropriateness of lower extremity MRI indications. They must be interpreted with caution given the SNDS limitation (mainly the lack of clinical data). It should be noted that we were unable to study the reason for requesting lower extremity MRI or to differentiate between traumatic and non-traumatic cases, as these data were not available in the SNDS. However, we excluded MRIs performed in emergency units in an attempt to focus on non-traumatic cases. In the case of repeated lower extremity MRIs, it is not possible to know whether the other lower extremity MRI is performed on the same segment of the lower extremity or even on the same side of the lower extremity as the index MRI. As a result, we may wrongly consider an examination as repeated, especially when examinations are close in time as it is unlikely that two MRIs are performed on the same part in a short period of time. Consequently, the proportion of patients who underwent a repeat MRI within a year is probably overestimated. We studied the proportion of patients who underwent a repeat MRI, considering only imaging procedures performed more than 7 days after the index MRI (Q1 of the period distribution between the index MRI and another MRI). The result is then 10.4% (versus 13.3% without a lag in the follow-up period). Nevertheless, in the absence of clinical data in the SNDS, it is difficult to differentiate appropriate practice (e.g., need for additional information due to changes in symptomatology) and irrelevant MRI (unjustified follow-up examination). Another limitation is that we chose to not include care performed on the day of the index MRI in the pre- and post-MRI follow-up, as it was not possible to know whether this care had been performed before or after the index MRI. Furthermore, the pre- and post-MRI follow-up was spread over 6 months before and after the index MRI, which may lead to an underestimation of care performed and should lead to a cautious interpretation of the results. However, this 6 months follow-up is consistent with the average time taken to access MRI in France [1], and to access surgical procedures in European countries [20].

Furthermore, this descriptive study did not have the objective to analyze the determining factors of inappropriate MRI. Analyzing whether specific age groups, genders, or other demographic factors are more likely to receive inappropriate MRIs could inform targeted educational or policy interventions. In the same way, analyzing the appropriateness rate of MRIs according to different categories of physicians could inform which physician to target. It can be noted that the physician requesting the MRI is the GP for 70.5% of the patients in our study. Although this proportion may be overestimated, we can assume that the GP is the requesting physician in at least half the cases, since in the 3 months prior to the index MRI, half the patients (52.2%) had at least one consultation with a GP, with no consultation with another medical specialty. Pending analysis of the key factors of inappropriate requests for lower extremity MRI, one of the first targets for intervention could be the GP.

Finally, it was not possible to analyze imaging practices in specific cases of knee pain. In fact, in the SNDS, only X-ray and orthopedic procedures are specific to the knee; the other imaging and outpatient procedures studied here do not allow us to distinguish the precise location of the lower extremities. We found no French data in the literature on the proportion of knee MRI among lower extremity MRI. We have performed a request on a single French hospital database and found that knee MRI represented 59% of lower extremity MRI. Nevertheless, this data does not represent the MRI practices at the national level. However, according to clinicians’ feedback on their practice, lower extremity MRI mainly concerns the knee. Subsequently, even if these results need to be tempered by the available data, they encourage us to disseminate the messages on the appropriateness of imaging in cases of knee pain published by HAS and G4 in 2022 to physicians requesting MRI (GPs in particular), but also to radiologists. We hope that the results of this study will contribute to a change in imaging practices, especially in case of knee pain. It would be interesting to evaluate these changes in a few years’ time.

## Supplementary information

ELECTRONIC SUPPLEMENTARY MATERIAL

## Abbreviations

GP: General practitioner
HAS: Haute Autorité de Santé (National Authority for Health)
MRI: Magnetic resonance imaging
PMR: physical medicine and rehabilitation
SNDS: Système National des Données de Santé (National Health Data System)
