# Supplementary material for: Lower extremity MRI: are their requests always appropriate in France?
Source: Eur Radiol. 2025 Feb 4;35(8):4692–8. doi: 10.1007/s00330-025-11402-w (PMC12226645; doi:10.1007/s00330-025-11402-w)
Supplement: Supplementary file 1 — ELECTRONIC SUPPLEMENTARY MATERIAL [file 330_2025_11402_MOESM1_ESM.pdf]

Lower extremity MRI: are their requests always appropriate in France?

ELECTRONIC SUPPLEMENTARY MATERIAL

Medical procedure	Code (standard)		Wording (in French)
	(B2)	(PS5)	
Consultation		1098	CONSULTATION CCMU 3
		1099	CONSULTATION CCMU 4 ET 5
	APU	1101	AVIS PONCTUEL DE CONSULTANT PUPH
	APY	1102	AVIS PONCTUEL DE CONSULTANT PSYCHIATRE
	APC	1103	AVIS PONCTUEL DE CONSULTANT DU MEDECIN
	CCX	1105	CONSULTATION COMPLEXE
	GS	1109	CONSULTATION SPECIALISTE MEDECINE GENERALE
	G	1110	CONSULTATION MEDECINE GENERALE
	C	1111	CONSULTATION COTEE C
	CS	1112	CONSULTATION COTEE CS
	CNP	1113	CONSULTATION COTEE CNP
	CSC	1114	CONSULTATION SPECIFIQUE CARDIOLOGIE
	CA	1115	CONSULTATION BILAN
		1117	CONSULTATION DES SPECIALISTES COTEE C2
		1118	CONSULTATION DES PSYCHIATRES COTEE C2,5
	CDE	1140	CONSULTATION SPECIFIQUE DE DEPISTAGE
	CCP	1168	CONSULTATION DE CONTRACEPTION ET PREVENTION
	TTE	1096	TELECONSULTATION MEDECIN TRAITANT AVEC EHPAD
	TCP	1157	ACTE DE TELECONSULTATION
	TLC	1164	TÉLÉ CONSULTATION - ALD ET / OU EHPAD
	TC	1191	TELECONSULTATION TOUTES SPECIALITES
	TCG	1192	TELECONSULTATION GENERALISTE
	(NABM)		
Knee X-ray	NFQK001		Radiographie unilatérale du genou selon 1 ou 2 incidences
	NFQK002		Radiographie bilatérale du genou selon 1 ou 2 incidences par côté
	NFQK003		Radiographie du genou selon 3 ou 4 incidences
	NFQK004		Radiographie du genou selon 5 incidences ou plus
Lower extremity X-ray except knee X-ray (pelvis, hip-joint, thigh, leg, ankle or foot X-ray)	NAQK015		Radiographie de la ceinture pelvienne [du bassin] selon 1 incidence
	NAQK007		Radiographie de la ceinture pelvienne [du bassin] selon 2 incidences
	NAQK023		Radiographie de la ceinture pelvienne [du bassin] selon 3 incidences ou plus
	NAQK071		Radiographie de la ceinture pelvienne [du bassin] selon 1 incidence et radiographie unilatérale de l'articulation coxofémorale selon 1 ou 2 incidences
	NAQK049		Radiographie de la ceinture pelvienne [du bassin] selon 1 incidence et radiographie bilatérale de l'articulation coxofémorale selon 1 ou 2 incidences par côté
	NEQK010		Radiographie de l'articulation coxofémorale selon 1 ou 2 incidences
	NEQK035		Radiographie de l'articulation coxofémorale selon 3 incidences
	NEQK012		Radiographie de l'articulation coxofémorale selon 4 incidences ou plus
	NBQK001		Radiographie de la cuisse
	NCQK001		Radiographie de la jambe

	NGQK001<br>NGQK002	Radiographie de la cheville selon 1 à 3 incidences<br>Radiographie de la cheville selon 4 incidences ou plus
	NDQK001<br>NDQK002<br>NDQK003<br>NDQK004	Radiographie unilatérale du pied selon 1 à 3 incidences<br>Radiographie bilatérale du pied selon 1 à 3 incidences par côté<br>Radiographie du pied selon 4 incidences ou plus<br>Radiographie du pied selon 4 incidences ou plus, pour étude podométrique
Lower extremity MRI	NZQN001<br>NZQJ001	Remnographie [IRM] unilatérale ou bilatérale de segment du membre inférieur, sans injection de produit de contraste<br>Remnographie [IRM] unilatérale ou bilatérale de segment du membre inférieur, avec injection de produit de contraste
Lower extremity CT-Scan	NZQH001<br>NZQK002	Scanographie unilatérale ou bilatérale de segment du membre inférieur, avec injection de produit de contraste<br>Scanographie unilatérale ou bilatérale de segment du membre inférieur, sans injection de produit de contraste
Joint ultrasound	PBQM001<br>PBQM002<br>PBQM003<br>PBQM004	Échographie unilatérale ou bilatérale de plusieurs articulations<br>Échographie unilatérale ou bilatérale de l’articulation<br>Échographie unilatérale ou bilatérale d’une articulation et de son appareil capsuloligamentaire<br>Échographie unilatérale ou bilatérale de plusieurs articulations et de leur appareil capsuloligamentaire
Interventionnel procedures (immobilization, puncture, drainage, infiltration)	NFMP001<br>NFMP002<br>NZMP007	Confection d’une contention souple du genou<br>Confection d'une attelle de posture ou de mobilisation du genou<br>Confection d'un appareil rigide d'immobilisation du membre inférieur prenant le genou
	NZHB002<br>NZHH004<br>NZHH001	Ponction ou cytoponction d'une articulation du membre inférieur, par voie transcutanée sans guidage<br>Ponction ou cytoponction d'une articulation du membre inférieur, par voie transcutanée avec guidage radiologique<br>Ponction ou cytoponction d'une articulation du membre inférieur, par voie transcutanée avec guidage scanographique
	NZJB001	Évacuation de collection articulaire du membre inférieur, par voie transcutanée sans guidage
	NZLB001<br>NZLH002<br>NZLH001	Injection thérapeutique d’agent pharmacologique dans une articulation ou une bourse séreuse du membre inférieur par voie transcutanée sans guidage<br>Injection thérapeutique d'agent pharmacologique dans une articulation ou une bourse séreuse du membre inférieur, par voie transcutanée avec guidage radiologique<br>Injection thérapeutique d'agent pharmacologique dans une articulation ou une bourse séreuse du membre inférieur, par voie transcutanée avec guidage scanographique
	(GHM root)	
Hospitalisation for knee intervention	08C45<br>08C34<br>08C38<br>08C24<br>08C53<br>08C54	Méniscectomie sous arthroscopie<br>Interventions sur les ligaments croisés<br>Autres arthroscopies du genou<br>Prothèses de genou<br>Interventions sur le genou pour traumatismes<br>Interventions sur le genou pour des affections autres que traumatiques
